# Retraction Note: STMN-1 is a potential marker of lymph node metastasis in distal esophageal adenocarcinomas and silencing its expression can reverse malignant phenotype of tumor cells

**DOI:** 10.1186/s12885-015-1162-8

**Published:** 2015-03-26

**Authors:** Javed Akhtar, Zhou Wang, Che Yu, Chen-Sheng Li, Yu-Long Shi, Hong-Jun Liu

**Affiliations:** 1 Department of Thoracic Surgery, Provincial Hospital Affiliated to Shandong University, Shandong, 250021 China; 2 Division of Gastrointestinal Surgery, Provincial Hospital Affiliated to Shandong University, Shandong, 250021 China; 3 Department of Nephrology, Provincial Hospital Affiliated to Shandong University, Shandong, 250021 PR China

## Retraction

The Publisher and Editor regretfully retract this article [1] because the peer-review process was inappropriately influenced and compromised. As a result, the scientific integrity of the article cannot be guaranteed. A systematic and detailed investigation suggests that a third party was involved in supplying fabricated details of potential peer reviewers for a large number of manuscripts submitted to different journals. In accordance with recommendations from COPE we have retracted all affected published articles, including this one. It was not possible to determine beyond doubt that the authors of this particular article were aware of any third party attempts to manipulate peer review of their manuscript.
